# Triplet Deep Hashing with Joint Supervised Loss Based on Deep Neural Networks

**DOI:** 10.1155/2019/8490364

**Published:** 2019-10-09

**Authors:** Mingyong Li, Ziye An, Qinmin Wei, Kaiyue Xiang, Yan Ma

**Affiliations:** College of Computer and Information Science, Chongqing Normal University, Chongqing 401331, China

## Abstract

In recent years, with the explosion of multimedia data from search engines, social media, and e-commerce platforms, there is an urgent need for fast retrieval methods for massive big data. Hashing is widely used in large-scale and high-dimensional data search because of its low storage cost and fast query speed. Thanks to the great success of deep learning in many fields, the deep learning method has been introduced into hashing retrieval, and it uses a deep neural network to learn image features and hash codes simultaneously. Compared with the traditional hashing methods, it has better performance. However, existing deep hashing methods have some limitations; for example, most methods consider only one kind of supervised loss, which leads to insufficient utilization of supervised information. To address this issue, we proposed a triplet deep hashing method with joint supervised loss based on the convolutional neural network (JLTDH) in this work. The proposed method JLTDH combines triplet likelihood loss and linear classification loss; moreover, the triplet supervised label is adopted, which contains richer supervised information than that of the pointwise and pairwise labels. At the same time, in order to overcome the cubic increase in the number of triplets and make triplet training more effective, we adopt a novel triplet selection method. The whole process is divided into two stages: In the first stage, taking the triplets generated by the triplet selection method as the input of the CNN, the three CNNs with shared weights are used for image feature learning, and the last layer of the network outputs a preliminary hash code. In the second stage, relying on the hash code of the first stage and the joint loss function, the neural network model is further optimized so that the generated hash code has higher query precision. We perform extensive experiments on the three public benchmark datasets CIFAR-10, NUS-WIDE, and MS-COCO. Experimental results demonstrate that the proposed method outperforms the compared methods, and the method is also superior to all previous deep hashing methods based on the triplet label.

## 1. Introduction

In recent years, because of the explosive growth of Internet big data, the Internet is filled with a large number of multimedia resources, including pictures, videos, and text, and there is an urgent need for fast search methods for massive big data. Approximate nearest neighbor (ANN) [1] search has been widely used in many fields such as image retrieval, computer vision, and data mining. Because of speed and low memory cost, hashing has become an important branch of ANN search, which is one of the widely used techniques in image retrieval [2–9]. Hashing techniques encode images, documents, videos, or other types of data in a short set of binary codes while keeping the original data similar. The hashing method produces binary encodings that make the nearest neighbor search of large datasets easy.

A series of different hashing methods are proposed to implement efficient ANN search using Hamming distance [3, 5, 8, 9]. More recently, deep hashing methods [10, 11, 12, 13, 14] show that image representation and hash coding can be learned more effectively using deep neural networks, resulting in state-of-the-art results on many benchmark datasets.

Recently, triplet loss has been studied for computer vision problems. The triplet labels contain richer information than pairwise labels. Each triplet label can be naturally decomposed into two pairwise labels. In particular, a triplet label ensures that the query image is close to the positive image and far away from the negative image in the learning hash code space. However, a pairwise label only ensures that one constraint is observed. The retrieval performance of triplet loss is better than that of pointwise and pairwise losses. Therefore, the triplet likelihood loss is introduced in this paper.

At the same time, the existing deep hashing methods still have some shortcomings in the utilization of classification information. The classification information only plays a role in deep neural network image representation but has no direct impact on the optimization of the hash function. Therefore, this paper proposes a linear classification loss to deal with this situation.

Therefore, combining triplet likelihood loss and linear classification loss, we propose a triplet deep hashing method with joint supervised loss based on the convolutional neural network (JLTDH). The supervised information used in this method is in the form of triplet labels. For the sake of fully utilizing the triplet information and the classification information, we propose a joint loss function, which consists of two parts: the triplet negative log-likelihood loss and the linear classification loss. Depending on this joint loss function, the hash codes can be further optimized by the linear classifier. The linear classifier indicates the relationship between the label information and the hash codes. We choose the convolutional neural network (CNN) as our deep learning model, for example, AlexNet, ResNet, and VGG, which can learn image representation and hash function at the same time. The whole process is divided into two stages: In the first stage, taking the triplets generated by the triplet selection method as the input of the CNN, the three CNNs with shared weights are used for image feature learning, and the last layer of the network outputs a preliminary hash code. In the second stage, relying on the hash code of the first stage and the joint loss function, the neural network model is further optimized so that the generated hash code has higher query precision.

This work is summarized as follows:

1. In this paper, a triplet deep hashing method with negative log-likelihood is proposed, and the method performs both image feature representation and hash code learning in a convolutional neural network. In order to overcome the cubic increase in the number of triplets and make triplet training more effective, we adopt a novel triplet selection method.
2. To fully utilize the supervised triplet information, JLTDH proposed a joint loss function combining the triplet negative log-likelihood loss and the linear classification loss. Relying on the hash code of the first stage and the joint loss function, the neural network model is further optimized so that the generated hash code has higher query precision.
3. We perform extensive experiments on the three public benchmark datasets CIFAR-10, NUS-WIDE, and MS-COCO. Experimental results demonstrate that the proposed method outperforms the compared methods, and the method is also superior to all previous deep hashing methods based on the triplet label.

## 2. Related Works

Hashing methods are divided into data-independent methods and data-dependent methods. Locality-sensitive hashing (LSH) [1] is a typical representative of data-independent hashing methods among various hashing techniques; the hash function of this method is generated by a random map, and random projection maps data points from the original space to the Hamming space; in this process, the training data are not used to learn hash functions. One drawback of LSH is that LSH usually requires a very long bit length, which leads to huge storage overhead. Data-dependent methods [15] learn the data feature from the training data so as to learn the hash function, which are also known as learning to hashing (L2H) methods. Compared to the data-independent method, the L2H method can get better accuracy with a shorter hash code. Therefore, the L2H method is more widely used than the data-independent method in the practical application.

The L2H methods include two types: unsupervised hashing and supervised hashing [4, 16]. Unsupervised hashing does not use supervised information for hash function learning, and the purpose of unsupervised hashing is to preserve the metric structure in the training data. Typical unsupervised approaches include iterative quantization (ITQ) [3], isotropic hashing (IsoHash) [4], discrete graph hashing (DGH) [15], and scalable graph hashing (SGH) [17]. Unsupervised hashing often fails to achieve satisfactory retrieval performance in practical applications.

On the contrary, supervised hashing tries to learn the hash function by utilizing supervised information. The purpose of supervised hashing is to map the data points in the original space to the Hamming space with supervised information, and supervised information optimizes the learning of hash function so as to learn better hash code. In recent years, using supervised information for learning, supervised hashing has higher precision than unsupervised hashing, so more and more researchers have studied it deeply [5, 12, 18]. Typical supervised hashing methods include supervised hashing with kernels (KSH) [5], fast supervised hashing (FastH) [8], supervised discrete hashing (SDH) [9], latent factor hashing (LFH) [19], adaptive binary quantization (ABQ) [20], hash bit selection [21], and structure-sensitive hashing (SSH) [10]. ABQ [20] jointly pursues a set of prototypes in the original space and a subset of binary codes in the Hamming space. The prototypes and the codes are correspondingly associated and together define the hash function for small hash codes. Hash bit selection [21] presented two related selection methods via dynamic programming and quadratic programming, incorporating bit reliability and complementarity. SSH [10] simultaneously captures the two types of structures among data in an alternative way. It learns discriminative hash functions that quantize data into the cluster prototypes associated with unique binary codes.

However, most traditional supervised hashing methods cannot extract features very well. In recent years, researchers have proposed deep learning hashing methods, which can effectively extract image features to identify similar images, and their performance is better than that of the traditional hashing method. Representative deep hashing methods including convolutional neural network-based hashing (CNNH) [18] adopt a two-stage strategy: learning binary hash codes in the first stage and learning a deep-network-based hash function to fit the codes in the second stage. DNNH [12] improved CNNH with a simultaneous feature learning and hash coding pipeline such that deep representations and hash codes are optimized by the triplet loss. The deep hashing network (DHN) [14] improves DNNH by jointly preserving the pairwise semantic similarity and controlling the quantization error by simultaneously optimizing the pairwise cross-entropy loss and quantization loss via a multitask approach. Other typical deep hashing methods include deep pairwise supervised hashing (DPSH) [13] and deep supervised hashing (DSH) [22].

Recently, some new deep hashing methods emerged, such as cross-modal hashing and hashing-based generative adversarial network. Representative methods including progressive generative hashing (PGH) [23] learn a discriminative hashing network in an unsupervised way, which exploits the power of hash-conditioned GANs and progressive learning. Triplet-based deep hashing (TDH) [24] is used for cross-modal retrieval, and triplet labels are exploited as supervised information to capture relative semantic correlation between heterogeneous data from different modalities. UCH [25] is a cross-modal retrieval method, where the outer-cycle network is used to learn powerful common representation and the inner-cycle network is explained to generate reliable hash codes. Deep learning hashing methods can significantly outperform nondeep supervised hashing in many applications, so we focused on deep hashing.

## 3. The Framework of JLTDH

### 3.1. Problem Definition

In our proposed method JLTDH, the input of the convolutional neural network is triplets. We denote the image triplet set as *T*={*t*_*i*_}_*i*=1_^*m*^={(*t*_*i*_^*a*^, *t*_*i*_^*p*^, *t*_*i*_^*n*^)}_*i*=1_^*m*^; in each triplet of the image *t*_*i*_=(*t*_*i*_^*a*^, *t*_*i*_^*p*^, *t*_*i*_^*n*^), *t*_*i*_^*a*^, *t*_*i*_^*p*^,  and *t*_*i*_^*n*^ indicate, respectively, the anchor point, the positive point, and the negative point; the positive image *t*_*i*_^*p*^ is defined as *s*_*ap*_=1 (*t*_*i*_^*p*^ and *t*_*i*_^*a*^ are similar and belong to the same category), and the negative image *t*_*i*_^*n*^ is defined as *s*_*an*_=0 (*t*_*i*_^*n*^ and *t*_*i*_^*a*^ are dissimilar and belong to different categories). The distance between *t*_*i*_^*a*^ and *t*_*i*_^*p*^ is smaller than the distance between *t*_*i*_^*a*^ and *t*_*i*_^*n*^.

Our goal is to learn the hash codes *B*={*b*_*i*_}_*i*=1_^*n*^ ∈ {−1,1}^*L*×*n*^ for the image *X*; the similarity between the two hash codes is calculated using the Hamming distance. The hash codes *B* should satisfy all the triplet labels *T* in the Hamming space as much as possible; for triplet labels, dis*H*(*b*_*t*_*i*_^*a*^_, *b*_*t*_*i*_^*p*^_) should be as small as possible as dis*H*(*b*_*t*_*i*_^*a*^_, *b*_*t*_*i*_^*n*^_), where dis*H*() represents the Hamming distance between two hash codes.

### 3.2. Framework

We introduce the proposed framework of JLTDH; this is an end-to-end hash learning framework based on the convolutional neural network.

**Figure 1:**
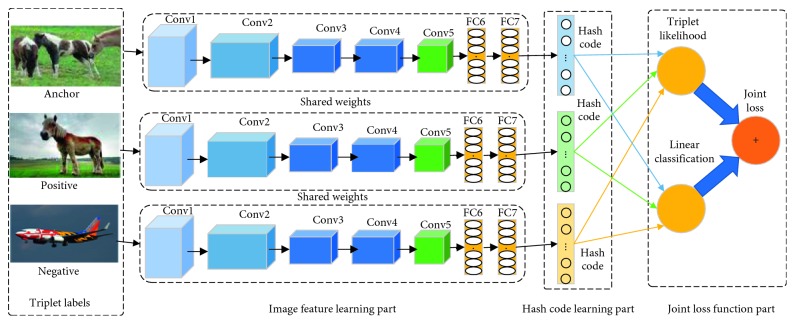
Framework of JLTDH.

As shown in Figure 1, we proposed a triplet deep hashing with joint supervised loss, which is a deep learning framework capable of both automatic feature learning and hash coding, and it joins triplet deep learning and linear classification quantization. This is an end-to-end approach and supervised by triplet labels, which contains three main components: image feature learning part, hash code learning part, and joint loss function part. It integrates these three components into the same end-to-end framework.

We generally generate triplets based on the category information of the sample, select the anchor image and positive image from the same category, and select the negative image from different categories. However, as the dataset increases, the number of all possible triplets is very large; using all triplets is computationally difficult and not optimal, and at the same time, it is not helpful for training and will lead to slow convergence of training. The existing triplet hashing method does not solve the problem of triplet selection very well. Therefore, the mining and selection of triplets is an urgent problem to be solved. We adopt a novel triplet selection method, which will be discussed in detail in Section 4.

#### 3.2.1. Image Feature Learning Part

In this part, we use three CNNs with shared weights to extract the appropriate feature representation for binary hash code learning. We use the AlexNet [26] network architecture for this part, and VGG [27] and ResNet [28] can also apply to this part as well. Each CNN contains five convolutional layers and three fully connected layers. The last layer of AlexNet is replaced with the FC (fully connected) layer, and the output of the last layer is projected as a hash code. The configuration of AlexNet is shown in Table 1.

#### 3.2.2. Hash Code Learning Part

This part generates the hash code of the image according to the image features of the previous part. The FC layer uses the sign functions as activation functions. Binary code is obtained by using the sign function. The length of the hash code *c* is determined by the number of FC-layer neurons in the last layer.

#### 3.2.3. Joint Loss Function Part

The joint loss function combines two kinds of supervised loss functions: the triplet negative log-likelihood loss and the linear classification loss, and is designed to further optimize the hash code so that the hash code and classification information can maintain the semantic relationship between points. The joint loss function is the weighted combination of triplet label likelihood loss and linear classification loss.

## 4. Triplet Selection Method

In large datasets like NUS-WIDE and MS-COCO, the number of all possible triplets is very large. Thus, using all triplets is computationally difficult and not optimal. Specifically, it is not helpful for training and will lead to slow convergence of training. For example, the dataset MS-COCO is used in this paper, whose training dataset contains 10,000 images, and the number of all possible triplets is approximately (1.0 × 10^4^)^3^=1.0 × 10^12^. This is a very large number which is very difficult to calculate.

Inspired by the study in [29], we adopt a novel triplet selection method to reduce the computational cost. Randomly splitting the training data into several groups {*G*_*i*_}_*i*=1_^*m*^, the triplets are selected only within groups *G*_*i*_^*T*^={*G*_*i*_^*a*^, *G*_*i*_^*p*^, *G*_*i*_^*n*^}, where *G*_*i*_^*a*^, *G*_*i*_^*p*^,  and *G*_*i*_^*n*^, respectively, represent the anchor points, positive points, and negative points in the *i*‐th group. *G*_*i*_^*p*^={*p* ∈ *G*_*i*_ :  *p* ≠ *a*, *s*_*pa*_=1} is the group of positive samples consisting of the samples similar to the anchor point *G*_*i*_^*a*^ in the *i*‐th group. We randomly chose negative samples from the group of negative samples *G*_*i*_^*n*^={*n* ∈ *G*_*i*_ :  *α* − dist(*a*, *n*)+dist(*a*, *p*) > 0, *s*_*an*_=0}, and we found that negative points far away from the anchor point were not helpful for training, so we excluded these negative points.

Using the proposed triplet selection method, we find that the number of triplets is much smaller than the number of possible triplets in our dataset. The specific results are shown in Table 2. The CPU running the code of the triplet selection method is Intel Xeon CPU E5-2687W @ 3.0 GHz with 12 cores, and the RAM is 32 GB. The time cost of the triplet selection method on three datasets is low and acceptable.

## 5. Joint Loss Function

The joint loss function combines two kinds of supervised loss functions: the triplet negative log-likelihood loss and the linear classification loss. We introduce them in the following.

### 5.1. Triplet Negative Log-Likelihood Loss

The Hamming distance between two strings of equal length is the number of positions at which the corresponding symbols are different. It is often used to calculate the similarity between two hash codes. In other words, the smaller the Hamming distance between two hash codes, the more similar they are, and vice versa. dist*H*(*b*_*i*_, *b*_*j*_) indicates the Hamming distance between *b*_*i*_ and *b*_*j*_, which can be calculated by inner product 〈*b*_*i*_, *b*_*j*_〉: dist*H*(*b*_*i*_, *b*_*j*_)=1/2(*L* − 〈*b*_*i*_, *b*_*j*_〉), where *L* is generally the length of the hash code. From the above equation, it can be concluded that the larger the inner product of the two hash codes, the smaller the Hamming distance and the more similar they are. Let *Ω*_*ij*_ represent half of the inner product between two binary codes *b*_*i*_, *b*_*j*_ ∈ {−1,1}^*L*^: *Ω*_*ij*_=1/2*b*_*i*_^*T*^ *b*_*j*_. DPSH [13] is a method for simultaneously learning feature representation and hash codes using pairwise label likelihood function as the loss function, and the likelihood function is formulated as

(1) $$\begin{array}{c} p\left(S\left|B\right.\right)=\underset{s_{ij}\in S}{\prod }p\left(s_{ij}\left|B\right.\right), \end{array}$$

where $p\left(s_{ij}\left|B\right.\right)=\left\{\begin{array}{cc} \varphi \left(\Omega_{ij}\right), & s_{ij}=1,\\ 1-\varphi \left(\Omega_{ij}\right), & s_{ij}=0, \end{array}\right.$ *B* is the set of all hash codes and *φ*(*x*) is the sigmoid function:*φ*(*x*)=1/1+*e* ^−*x*^. The two hash codes with the large inner product should have high similarity.

The supervised information used by DPSH [13] is a pairwise label. Inspired by DPSH, this paper proposes a hashing method using triplet label likelihood function, which is a deep hashing method using a deep neural network. A set of triplet labels *T*={*t*_*i*_}_*i*=1_^*m*^={(*t*_*i*_^*a*^, *t*_*i*_^*p*^, *t*_*i*_^*n*^)}_*i*=1_^*m*^ is given. Suppose the conditions are independent of each other, according to naive Bayes' theorem, with some prior *p*(*B*), then the posterior probability of *B* can be computed as follows:

(2) $$\begin{array}{c} p\left(B\left|T\right.\right)∼p\left(T\left|B\right.\right)p\left(B\right)=\underset{t_{i}\in T}{\prod }p\left(t_{i}\left|B\right.\right)p\left(B\right). \end{array}$$

We can define the triplet label likelihood function *p*(*T*|*B*) as follows:

(3) $$\begin{array}{c} p\left(T\left|B\right.\right)=\underset{t_{i}\in T}{\prod }p\left(t_{i}\left|B\right.\right)=\underset{t_{i}\in T}{\prod }p\left(\left(t_{i}^{a},t_{i}^{p},t_{i}^{n}\right)\left|B\right.\right). \end{array}$$

We can learn the optimal *B* through maximum a posteriori estimation.

In order to solve *p*((*t*_*i*_^*a*^, *t*_*i*_^*p*^, *t*_*i*_^*n*^)|*B*), according to dist*H*(*b*_*i*_, *b*_*j*_)=1/2(*L* − 2*Ω*_*ij*_), we can get

(4) $$\begin{array}{c} \text{dis}H\left(b_{t_{i}^{a}},b_{t_{i}^{p}}\right)-\text{dis}H\left(b_{t_{i}^{a}},b_{t_{i}^{n}}\right)=-\left(\Omega_{t_{i}^{a}t_{i}^{p}}-\Omega_{t_{i}^{a}t_{i}^{n}}\right). \end{array}$$

From equation (4), we can conclude that the smaller the distance between the anchor point and the positive point, and the greater the distance between the anchor point and the negative point, the larger the value of *Ω*_*t*_*i*_^*a*^*t*_*i*_^*p*^_ − *Ω*_*t*_*i*_^*a*^*t*_*i*_^*n*^_.

According to equation (4), we can make the following definition:

(5) $$\begin{array}{c} \;p\left(\left(t_{i}^{a},t_{i}^{p},t_{i}^{n}\right)\left|B\right.\right)=\varphi \left(\Omega_{t_{i}^{a}t_{i}^{p}}-\Omega_{t_{i}^{a}t_{i}^{n}}-\beta \right), \end{array}$$

where *β* is a superparameter whose role is to adjust the gap between dis*H*(*b*_*t*_*i*_^*a*^_, *b*_*t*_*i*_^*p*^_) and dis*H*(*b*_*t*_*i*_^*a*^_, *b*_*t*_*i*_^*n*^_). When dis*H*(*b*_*t*_*i*_^*a*^_, *b*_*t*_*i*_^*n*^_) ≥ dis*H*(*b*_*t*_*i*_^*a*^_, *b*_*t*_*i*_^*p*^_)+*β*, we can conclude that *Ω*_*t*_*i*_^*a*^*t*_*i*_^*p*^_ − *Ω*_*t*_*i*_^*a*^*t*_*i*_^*n*^_ − *β* ≥ 0 and *p*((*t*_*i*_^*a*^, *t*_*i*_^*p*^, *t*_*i*_^*n*^)|*B*) ≥ 0.5. The smaller the distance between the anchor point and the positive point, and the greater the distance between the anchor point and the negative point, the larger the value of the triplet likelihood function, and vice versa. This is consistent with the intention of our objective function.

By maximizing the triplet likelihood *p*(*T*|*B*), we can enforce the Hamming distance between the anchor image and the positive image to be smaller than that between the anchor image and the negative image. Taking the negative log-likelihood of the triplet label, the following optimization problem can be obtained:

(6) J1Bmin=−log pTB=−∑ti∈Tlog ptia,tip,tinB.

The above optimization problem can minimize the Hamming distance between the anchor point and the positive point and simultaneously maximize the Hamming distance between the anchor point and the negative point. This exactly matches the goal of supervised hashing with the triplet label.

Substituting formula (5) into formula (6), the following formula can be obtained:

(7) J1Bmin=−log pTB=−∑ti∈Tlog φΩtiatip−Ωtiatin−β=−∑ti∈TΩtiatip−Ωtiatin−β−log1+eΩtiatip−Ωtiatin−β.

A common problem with the deep hashing methods is how to train the neural network output to be binary codes [11, 20, 25, 26]. Minimizing the loss of equation (7) is a very difficult discrete optimization problem [30]. The usual method is to relax {*b*_*i*_} from discrete to continuous. In the last layer of the neural network, we use the sign function as the activation function to obtain the binary code. However, the sign function is nondifferentiable because the gradient of the sign function always equals zero, and the backpropagation of the loss function is difficult to proceed. A good method is to relax the binary codes and add a quantization error term in the objective function during training. This method is also utilized in [30, 25].

This method is described below: we use {*u*_*i*_} to denote the continuous output of the last layer before the sign function for the image. {*b*_*i*_} can be obtained by  *b*_*i*_=sgn(*u*_*i*_). We relax {*b*_*i*_} to continuous vectors {*u*_*i*_}, and we redefine *Ω*_*ij*_ as follows:

(8) $$\begin{array}{c} \Phi_{ij}=\frac{1}{2}u_{i}^{T}u_{j}. \end{array}$$

Then, we approximate equation (7) as

(9) ltriplet=J2Bmin=−∑ti∈TΦtiatip−Φtiatin−β−log1+eΦtiatip−Φtiatin−β+η∑i=1Nbi−ui22,

where ∑_*i*=1_^*N*^‖*b*_*i*_ − *u*_*i*_‖_2_^2^ represents the quantization error term and *η* is the superparameter, which is used to balance the original objective function and quantization error.

To integrate the above image feature representation part and the loss function part into a deep neural network framework, we set

(10) $$\begin{array}{c} u_{i}=W^{T}\Theta \left(x_{i};\;\phi \right)+\upsilon , \end{array}$$

where *ϕ* stands for all the parameters of the neural network except for the last layer, Θ(*x*_*i*_;  *ϕ*) represents the output of the neural network, *W* represents a weight matrix, and *υ* is a bias term.

### 5.2. Joint Learning with Linear Classification Quantization Loss

Triplet label information was used to learn hash codes by equation (9), but the label information was not fully utilized. We hope to fully utilize the label information so that we use the joint learning linear classifier to further optimize the hash code, making the learned hash code optimal. Inspired by the study in [31], we use the following classifier, which can represent the relationship between the learned hash code *B* and label information *Y*:

(11) $$\begin{array}{c} Y=W^{T}B, \end{array}$$

where *W*_*L*×*c*_={*w*_*i*_}_*i*=1_^*c*^ is the classifier weight and *Y*_*c*×*N*_={*y*_*i*_}_*i*=1_^*N*^ is the ground-truth label vector, in which*c* is the number of categories in the dataset. We usually choose *l*2 loss for the linear classifier, and we define the loss function as follows:

(12) $$\begin{array}{c} l_{\text{linear}}=L\left(Y,W^{T}B\right)+\mu {\left\|W\right\|}_{F}^{2}=\overset{N}{\underset{i=1}{\sum }}{\left\|y_{i}-W^{T}b_{i}\right\|}_{2}^{2}+\mu {\left\|W\right\|}_{F}^{2}, \end{array}$$

where *l*_linear_ is the linear classifier loss function, *μ* is the regularization parameter, and ‖‖_*F*_^2^ is the *F* norm of a matrix. Equations (9) and (12) are combined by weight parameters, and the following formula can be obtained:

(13) $$\begin{array}{c} l_{\text{total}}=l_{\text{triplet}}+\lambda l_{\text{linear}}=-\underset{t_{i}\in T}{\sum }\left(\Phi_{t_{i}^{a}t_{i}^{p}}-\Phi_{t_{i}^{a}t_{i}^{n}}-\beta -\log\left(1+e^{\Phi_{t_{i}^{a}t_{i}^{p}}-\Phi_{t_{i}^{a}t_{i}^{n}}-\beta }\right)\right)\\ +\eta \overset{N}{\underset{i=1}{\sum }}{\left\|b_{i}-u_{i}\right\|}_{2}^{2}+\lambda \left(\overset{N}{\underset{i=1}{\sum }}{\left\|y_{i}-W^{T}b_{i}\right\|}_{2}^{2}+\mu {\left\|W\right\|}_{F}^{2}\right), \end{array}$$

where ‖‖_2_^2^ is the *l*2 norm of a vector and *λ* is the trade-off parameter used to balance the triplet likelihood term and the linear classification loss.

### 5.3. Optimization

Equation (13) is a joint loss optimization problem, which is still nonconvex, and it is difficult to solve. Here, we adopt the discrete cyclic coordinate descent (DCC) optimization method. Equation (13) can be decomposed into two suboptimization problems, and the linear classification loss can be solved iteratively by alternating minimization. For equation (12), the linear classification loss can be rewritten as

(14) $$\begin{array}{c} l_{\text{linear}}{=}_{B,W}^{\min}{\left\|Y-W^{T}B\right\|}_{2}^{2}+\mu {\left\|W\right\|}_{F}^{2}. \end{array}$$

By fixing *B*, using the matrix trace function tr(), the following formula is obtained:

(15) $$\begin{array}{c} \underset{W}{\min}{\left\|Y-W^{T}B\right\|}_{2}^{2}+\mu {\left\|W\right\|}_{F}^{2}=\underset{W}{\min}\text{tr}\left({\left(Y-W^{T}B\right)}^{T}\left(Y-W^{T}B\right)\right)+\mu \text{tr}\left(W^{T}W\right). \end{array}$$

Taking the derivative of *W* and setting *dJ*(*W*)/*dW*=0, we get the minimum value of *W*:

(16) $$\begin{array}{c} W={\left(BB^{T}+\mu I\right)}^{-1}BY^{T}. \end{array}$$

So once we solve *W*, we assume *W* as a constant matrix. By fixing *W*, equation (14) becomes

(17) $$\begin{array}{c} l_{\text{linear}}=\underset{B}{\min}{\left\|Y-W^{T}B\right\|}_{2}^{2}+\mu {\left\|W\right\|}_{F}^{2}=\underset{B}{\min}{\left\|Y\right\|}^{2}-2\text{tr}\left(Y^{T}W^{T}B\right)+{\left\|W^{T}B\right\|}^{2}. \end{array}$$

We can get a closed solution to a row of *B* by fixing the other rows. We use the discrete cyclic coordinate descent method to iteratively solve *B* row by row. Let *z* ^*T*^ be the *k* − th row of *B*, *k*=1,2, ..., *K* (*K* is the length of the hash code), and *B*′ the matrix of *B* excluding *z* ^*T*^. Let *v* ^*T*^ be the *k* − th row of *W* and *W*′ the matrix of *W*_12×10_ excluding *v* ^*T*^. The third term in equation (17) can be solved as follows:

(18) $$\begin{array}{c} {\left\|W^{T}B\right\|}^{2}={\left\|{\left[\begin{array}{c} W^{\prime }\\ v^{T} \end{array}\right]}^{T}\left[\begin{array}{c} B^{\prime }\\ z^{T} \end{array}\right]\right\|}^{2}={\left\|W^{\prime T}B^{\prime }+vz^{T}\right\|}^{2}={\left\|W^{\prime T}B^{\prime }\right\|}^{2}+{\left\|vz^{T}\right\|}^{2}+2tr\left({\left(W^{\prime T}B^{\prime }\right)}^{T}vz^{T}\right)\\ =\text{const}+2v^{T}W^{\prime T}B^{\prime }z. \end{array}$$

Similarly, let *Q*=*WY*, and let *q* ^*T*^ be the *k* ^th^ row of *Q* and *Q*′ the matrix of *Q* excluding *q* ^*T*^. The second term in equation (17) can be solved as follows:

(19) $$\begin{array}{c} \text{tr}\left(Y^{T}W^{T}B\right)=\text{tr}\left(B^{T}Q\right)=\text{tr}\left({\left[\begin{array}{c} B^{\prime }\\ z^{T} \end{array}\right]}^{T}\left[\begin{array}{c} Q^{\prime }\\ q^{T} \end{array}\right]\right)\\ =\text{tr}\left(B^{\prime }Q^{\prime }+zq^{T}\right)=\text{tr}\left(B^{\prime }Q^{\prime }\right)+\text{tr}\left(q^{T}z\right)=\text{const}+q^{T}z. \end{array}$$

Putting equations (17), (18), and (19) altogether, there is an optimal solution to this problem:

(20) $$\begin{array}{c} l_{\text{linear}}=\underset{B}{\min}{\left\|W^{T}B\right\|}^{2}-2\text{tr}\left(Y^{T}W^{T}B\right)=\underset{z}{\min}\left(v^{T}W^{\prime T}B^{\prime }-q^{T}\right)z \end{array}$$

(21) $$\begin{array}{c} z=\mathrm{sgn}{\left(q^{T}-v^{T}W^{\prime T}B^{\prime }\right)}^{T}=\mathrm{sgn}\left(q-B^{\prime T}W^{\prime }v\right). \end{array}$$

According to equation (21), each bit hash code *z* is calculated according to the *k* − 1 bit *B*′ that has been learned. We can iteratively update each bit until the program converges to a better set of hash codes *B*.

The proposed method JLTDH is briefly summarized in Algorithm 1.

## 6. Experiment and Analysis

In this section, we will describe our experiments and results. Three commonly used datasets are used to verify the effectiveness of our algorithm. We calculated the precision and mean average precision (MAP) of the retrieval results and showed the performance of our method on CIFAR-10, NUS-WIDE, and MS-COCO. Specifically, given an anchor *x*_*q*_, we can calculate its average precision (AP) using the following equation:

(22) $$\begin{array}{c} \text{AP}\left(x_{q}\right)=\frac{1}{R_{k}}\overset{n}{\underset{k=1}{\sum }}P\left(k\right)\Delta r\left(k\right), \end{array}$$

where *R*_*k*_ is the number of relevant samples, *P*(*k*) is the precision at cutoff *k* in the returned sample list, and Δ*r*(*k*) is an indicator function which equals 1 if the *k*-th returned sample is a ground-truth neighbor of *x*_*q*_. Otherwise, Δ*r*(*k*) is 0. Given *Q* queries, MAP is the AP of all query results sorted; we can compute the MAP as follows:

(23) $$\begin{array}{c} \text{MAP}=\frac{1}{Q}\overset{Q}{\underset{q=1}{\sum }}\text{AP}\left(x_{q}\right). \end{array}$$

### 6.1. Experimental Settings

Our server configuration is as follows: the CPU is Intel Xeon CPU E5-2687W @ 3.0 GHz with 12 cores, the GPU is NVIDIA GTX 1080 8 GB, and the RAM is 32 GB. The Linux operating system is Ubuntu 16.04, and the deep learning framework is PyTorch [32].

We use three widely used benchmark datasets of different scales; they are CIFAR-10 [33], NUS-WIDE [34], and MS-COCO [35]. The CIFAR-10 dataset contains 60,000 images and 10,000 test images, belonging to 10 categories. The size of each image is 32 × 32 pixels. We randomly selected 5,000 images (500 for each class) as the training set and 1,000 images (100 for each class) as the test query set.

The NUS-WIDE dataset contains 269,648 images in 81 categories. We used the 21 most commonly used categories. We randomly selected 2,100 images (100 images per class) as the query point and randomly selected 10,500 images (500 images per class) as the training set.

MS-COCO is an image dataset widely used for image recognition, segmentation, and captioning. It contains 82,783 training images and 40,504 validation images, in which each image is labeled by some of the 80 semantic concepts. We randomly selected 5,000 images as the query point and the rest images as the database and randomly sample 10,000 images from the database for training.  Table 3 shows some sample points from three datasets.  Table 4 shows the dataset settings used in our experiment.

We compared our method with several representative hashing methods for MAP; the comparison methods we selected are divided into two groups: traditional hashing methods and deep hashing methods. Traditional unsupervised hashing methods include SH [36] and ITQ [3]. Traditional supervised hashing methods include KSH [5], FastH [8], SDH [9], SPLH [37], and LFH [19]. The deep hashing methods include DSRH [30], DSCH [38], DRCSH [38], CNNH [18], NINH [12], DPSH [13], DHN [14], DQN [39], DTSH [40], VDSH [41], and DSDH [42]. In this paper, we also emphasize on the comparison of the deep hashing methods with triplet labels, including DSRH [37], DSCH [38], DRSCH [38], and NINH [12]. To be fair, some test results are directly evaluated in previously published papers. Following [41], CNN features on CIFAR-10 were extracted using the pretrained CNN-F model. The hyperparameters of this method are set according to the standard cross-validation procedure. In equation (12), *u* is set to 0.1, *λ* is 1, *η* is 55, and *β* is half the length of the hash code.

### 6.2. Empirical Analysis

#### 6.2.1. Comparison to Other Deep Methods and Nondeep Methods

**Figure 2:**
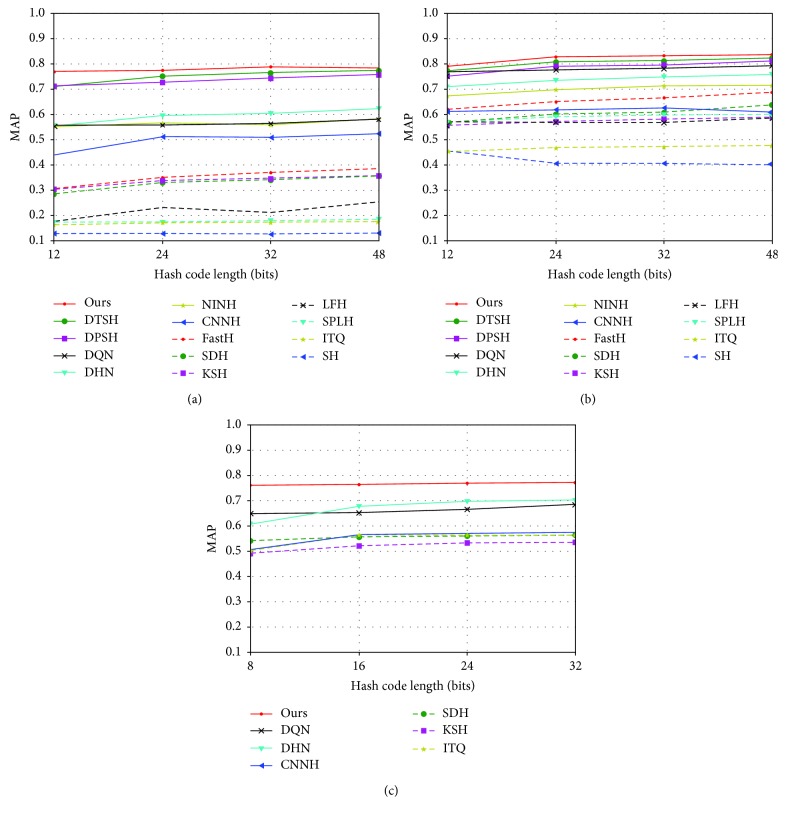
MAP results for different numbers of bits on the three benchmark image datasets. (a) CIFAR-10. (b) NUS-WIDE. (c) MS-COCO.

As shown in Table 5 and Figure 2, the MAP is calculated based on the top 5,000 returned neighbors. NINH, CNNH, KSH, and ITQ results were obtained from the study in [11, 18]. Results of other methods were obtained from the study in [42]. Our method performs better in these three datasets than the existing hashing methods; compared with nondeep learning methods, our method has been significantly improved. Our method further improved performance by 2–6% compared to the current best deep learning methods. At the same time, we found that our method performed better in shorter hash codes (≤32 bits). Most deep hashing methods have significant performance advantages.

MAP results for different numbers of bits on the MS-COCO dataset are shown in Table 6; except for our method, the other results were obtained from the study in [29]. In order to be consistent with the settings in [29], we set the hash code length as 8 bits, 16 bits, 24 bits, and 32 bits. The image pixel of MS-COCO is more complex, which will lead to more difficult classification, which may lead to inaccurate results of feature extraction and inaccurate classification results. As can be seen from Table 5, the performance of the MS-COCO dataset decreased to a certain extent compared with the results of MAP of the NUS-WIDE dataset. In spite of this, our method is still much better than the comparison methods.

**Figure 3:**
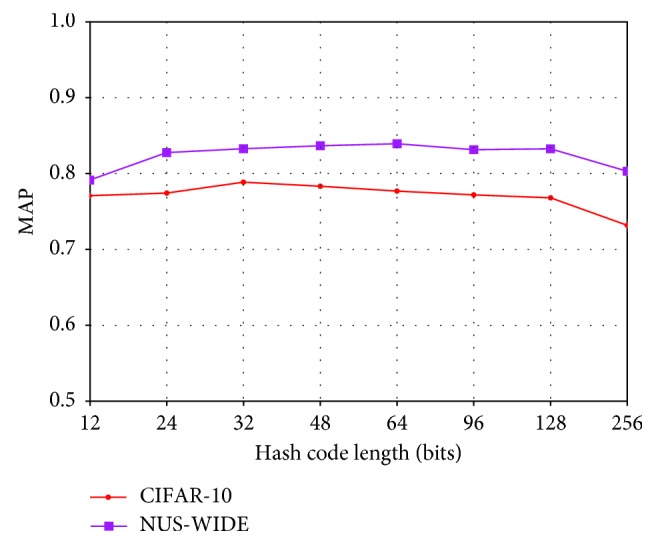
Effects of longer hash codes.

Our method can achieve excellent performance under shorter hash codes. At the same time, we also discussed the performance change under the long hash code. Since the comparison method does not provide the results of long hash codes, we only discuss our own method here. As can be seen from Figure 3, CIFAR-10 gets a good MAP value at 32 bits, while NUS-WIDE gets a good MAP value between 48 and 64 bits. With the growth of hash code length, the MAP of CIFAR-10 is decreasing, while the MAP of NUS-WIDE is not decreasing, but there is no obvious increase.

#### 6.2.2. Comparison to Nondeep Hashing Methods Using Deep Features

**Figure 4:**
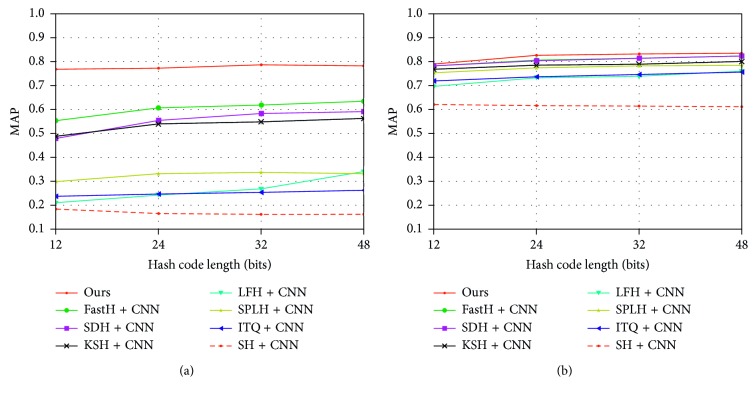
MAP results for different numbers of bits on the two benchmark image datasets (a) CIFAR-10 and (b) NUS-WIDE, compared to different traditional hashing methods using the CNN.

One might say that the performance improvements come from the neural network, not our approach. In order to further verify our method, we compared our method with the traditional hashing method using the CNN-F network [43] to extract the depth features. As shown in Figure 4, we can see that our method is significantly superior to the traditional method. It should be noted that most of the traditional methods are not tested based on MS-COCO, and we cannot get the corresponding data, so we did not do the comparative test of MS-COCO in this part.

#### 6.2.3. Comparison to Hashing Methods with Triplet Labels

**Figure 5:**
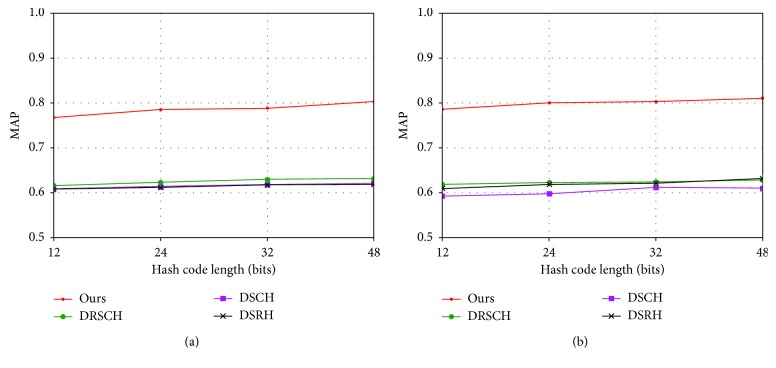
MAP results for different numbers of bits on the two benchmark image datasets (a) CIFAR-10 and (b) NUS-WIDE, compared with the triplet label methods.

This paper adopts the method of triplet labels; therefore, we focus on the comparison of the deep hashing methods with the triplet label, and we will compare it further with other deep hashing methods using the triplet label. These methods include DSRH [37], DSCH [38], DRSCH [38], and NINH [12]. The results of DSRH, DSCH, and DRSCH were adopted from the study in [38]. As shown in Figure 5, compared with previous deep hashing methods based on the triplet label, our method is obviously better and leads the comparative method by a wide margin.

### 6.3. Sensitivity to Hyperparameters

The most important hyperparameters for JLTDH are *λ* and *η*. *λ* is the trade-off parameter used to balance the triplet likelihood term and the linear classification loss. *η* is used to balance the likelihood term and the quantization error. We explore the influence of these two hyperparameters.

**Figure 6:**
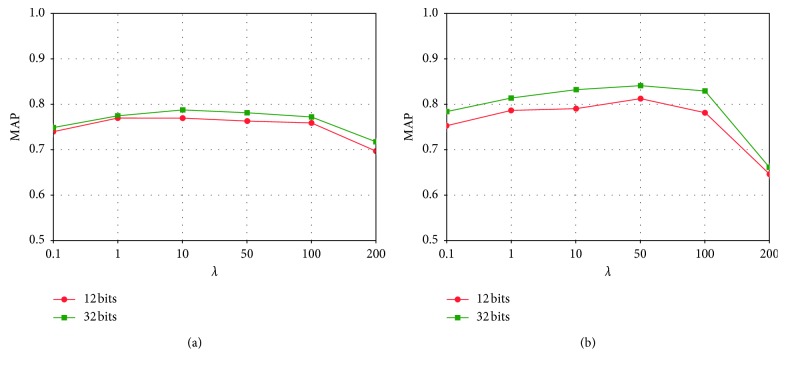
Effects of *λ* on (a) CIFAR-10 and (b) NUS-WIDE. Hash codes = 12 and 32 bits.

We report the MAP values for different *λ* from the range of [0.1, 200] on two datasets with the code length being 12 bits and 32 bits. We can find that JLTDH is not sensitive to *λ* in a large range when 10 < *λ* < 50. According to Figure 6(a), we found that when *λ*=10, CIFAR-10 can obtain the best MAP performance. As shown in Figure 6(b), when 10 < *λ* < 50, NUS-WIDE can achieve better MAP performance.

**Figure 7:**
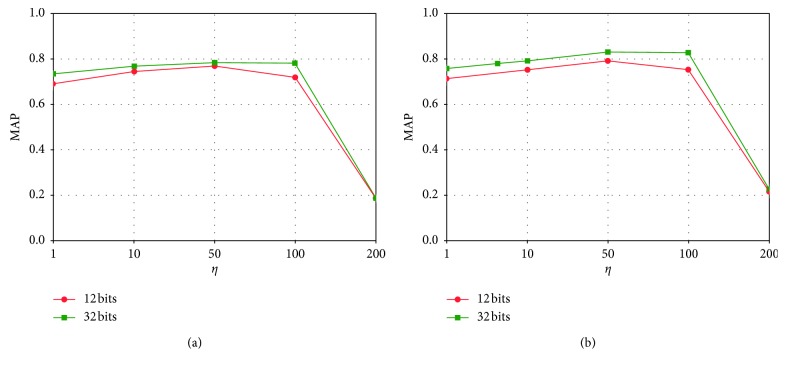
Effects of *η* on (a) CIFAR-10 and (b) NUS-WIDE. Hash codes = 12 and 32 bits.

Furthermore, we present the influence of *η* in Figures 7(a) and 6(b). As can be seen, there is a wide range of *η* in that our method performs well. When hash code = 12 bits, MAP accuracy is better on both datasets with *η*=50, and when hash code = 32 bits, MAP accuracy is better on both datasets with 50 ≤ *η* ≤ 100. Other superparameter settings are obtained through cross-validation: *u* is set to 0.1, and *β* is half the length of the hash code.

### 6.4. Analysis of Three Loss Functions

**Figure 8:**
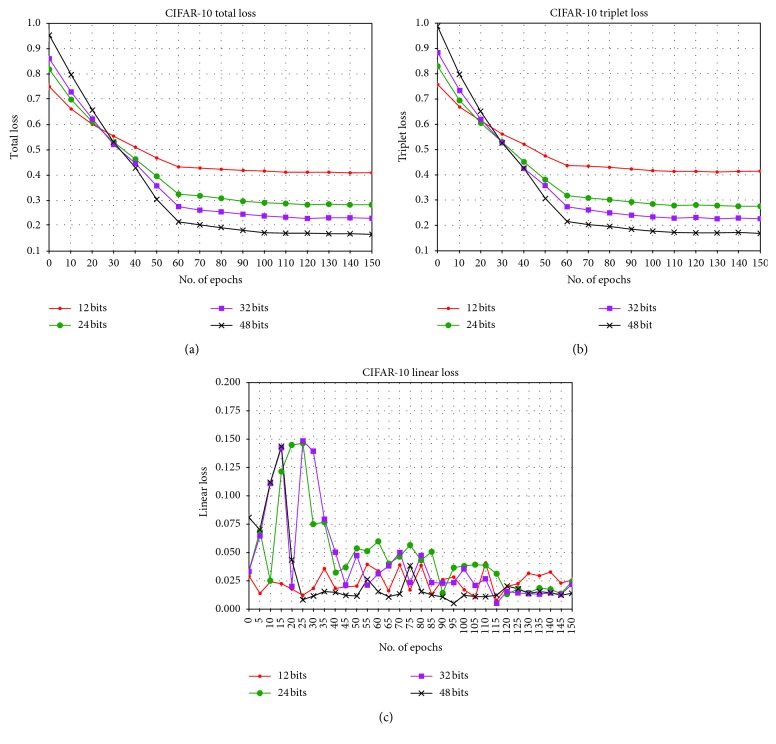
Convergence of three kinds of losses for different lengths of hash codes (epoch = 150). (a) Joint loss. (b) Triplet likelihood loss. (c) Linear classification loss.

By observing the convergence of the loss function, we can judge whether the selected loss function is reasonable or not.  Figure 8 shows the change of three kinds of losses for different lengths of hash codes during training. We only take CIFAR-10 as an example; the results for the other two datasets are similar. It is reasonable to conclude that the joint loss combining triplet likelihood loss and linear classification quantization loss successfully optimizes the loss during training. Figures 8(a) and 8(b) are similar: they show that the joint loss and triplet likelihood loss converge rapidly and are kept at a low level for different bits, that our method is reasonable and effective, and that the optimization process only needs a few iterations.

In order to further reveal the respective influence of the two parts of the joint loss function, we have shown the loss curves of triplet likelihood loss and linear classification loss, respectively, in Figures 8(b) and 8(c). The magnitude of the loss value in Figure 8(c) is about 10^−1^ of that in Figure 7(b), which is because triplet likelihood loss is used to optimize the first stage and plays a major role in training, while linear classification loss is used to optimize the second stage, which is further optimization based on the first stage and fine-tuning optimization.

#### 6.4.1. Ablation Study about Loss Function

**Figure 9:**
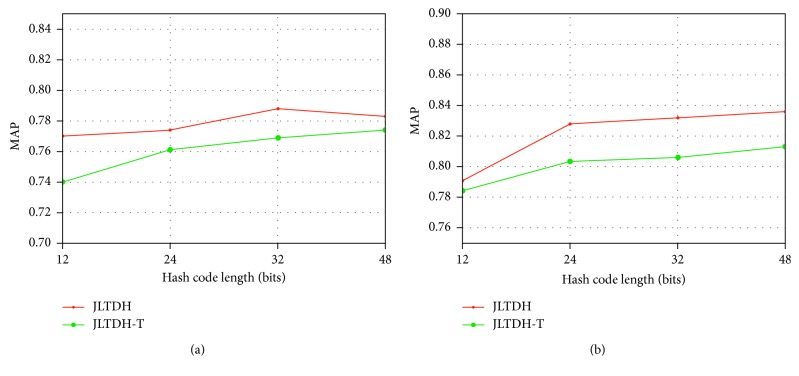
(a) Ablation effects of loss on CIFAR-10. (b) Ablation effects of loss on NUS-WIDE.

In order to confirm the contribution of different losses to final performance, we selected a variant of JLTDH for comparison: JLTDH-T is a variant of JLTDH, whose loss function contains only triplet loss and no linear classification loss. As can be seen from Figure 9, on the CIFAR and NUS-WIDE datasets, JLTDH-T can achieve good MAP performance with only triplet loss. Based on further optimization of linear classification loss, JLTDH achieves better MAP performance by using the joint loss. JLTDH is about 2% ahead of JLTDH-T on average.

### 6.5. Visualization of Query Results

**Figure 10:**
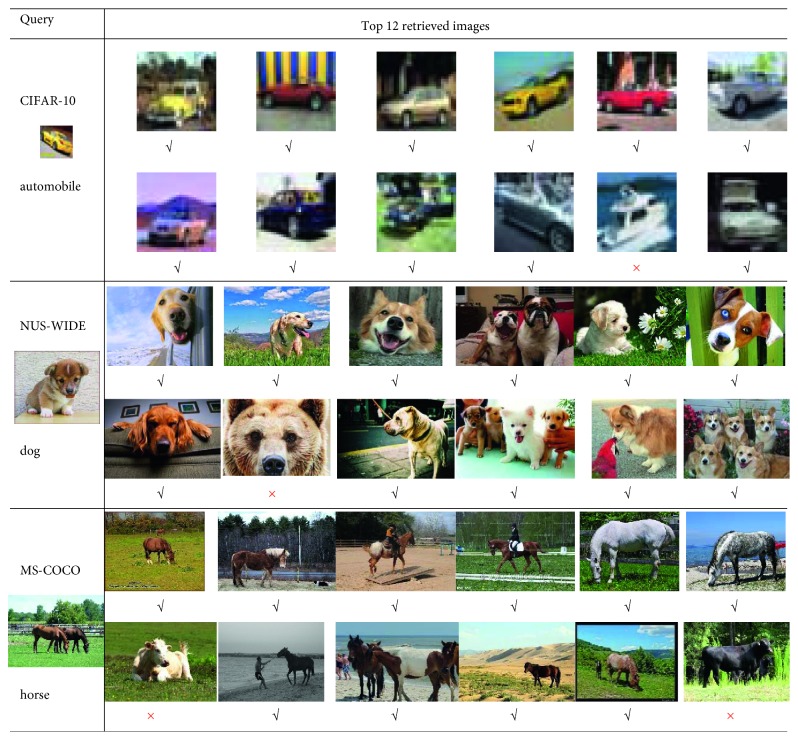
The top 12 images returned by the proposed method.

We show the illustration of top 12 returned images for better understanding of the impressive performance improvement of the proposed method.  Figure 10 illustrates the top 12 returned images of the proposed method for three query images on the three datasets CIFAR-10, NUS-WIDE, and MS-COCO, and the length of the hash code is 32. It shows that the method we proposed can truly preserve the features of an image and save them to the hash code. Regarding the query image in CIFAR-10, only one of the returned results is wrong, and the wrong image is only at the bottom of the sorted image. In contrast, for the query image in NUS-WIDE, only 1 out of the top 12 images is incorrect, and the top 12 images have a similar shape or similar color to the query image. And for the query image in MS-COCO, ten of the top 12 images are correct; compared with the previous two datasets, the query accuracy is slightly reduced. The possible reason is that MS-COCO is a multiobjective dataset. This shows that our method can provide the desired search results.

## 7. Conclusion

In this work, we propose a triplet deep hashing method with joint supervised loss based on the convolutional neural network (JLTDH). To fully utilize the supervised triplet information, this paper proposed a joint loss function combining two kinds of supervised loss functions: the triplet negative log-likelihood loss function and the linear classification loss function. At the same time, in order to overcome the cubic increase in the number of triplets and make triplet training more effective, we designed a triple selection method. The whole process is divided into two stages: Firstly, the last layer of the network outputs a preliminary hash code. Secondly, relying on the joint loss function and backpropagation algorithm, the parameters of the neural network are further updated so that the generated hash code has higher query precision. We perform extensive experiments on the three public benchmark datasets CIFAR-10, NUS-WIDE, and MS-COCO. Experimental results demonstrate that the proposed method outperforms the compared methods and is also superior to all previous deep hashing methods based on the triplet label.

## Figures and Tables

**Algorithm 1:**
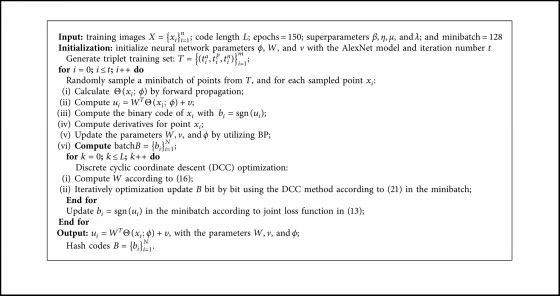
The procedure of JLTDH.

**Table 1:** Configuration of AlexNet in our method.

Layer	Configuration				
	#Filter	Filter size	Stride	Padding	Pooling
Conv1	64	11 × 11	4 × 4	2 × 2	3 × 3
Conv2	192	5 × 5	1 × 1	2 × 2	3 × 3
Conv3	384	3 × 3	1 × 1	1 × 1	—
Conv4	256	3 × 3	1 × 1	1 × 1	—
Conv5	256	3 × 3	1 × 1	1 × 1	3 × 3
Full6		9216			
Full7		4096			
Full8		Hash code length *c*			

**Table 2:** Number of triplets using the proposed triplet selection method.

Dataset	Training dataset	Group	Category	Selected triplets	Time
CIFAR-10	5000	20	10	124,000	86 s
NUS-WIDE	10,500	20	21	1,372,000	316 s
MS-COCO	10,000	20	80	1,719,000	492 s

**Table 3:** Examples of the datasets.

Dataset	Example	Label
CIFAR-10	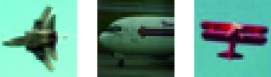	“airplane”
	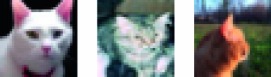	“cat”
	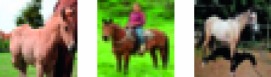	“horse”

NUS-WIDE	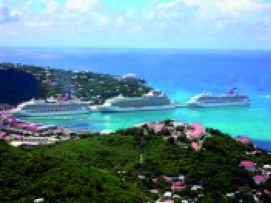	“clouds,” “ocean,” “sky,” “water”
	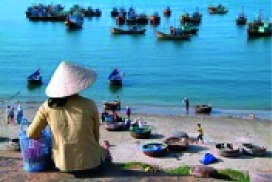	“person,” “ocean,” “water”

MS-COCO	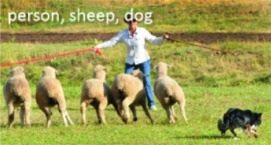	“person,” “sheep,” “dog”
	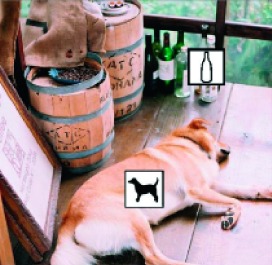	“dog,” “bottle,” “barrel”

**Table 4:** Dataset settings used in our experiment.

Dataset	Total	Training (randomly)	Testing (randomly)	Labels
CIFAR-10	60,000	5,000 (500 ∗ 10)	1,000 (100 ∗ 10)	10
NUS-WIDE	269,648	10,500 (500 ∗ 21)	2,100 (100 ∗ 21)	21
MS-COCO	82,783	10,000	5,000	80

**Table 5:** MAP results for different numbers of bits (12, 24, 32, and 48 bits) on the two benchmark image datasets (CIFAR-10 and NUS-WIDE).

Methods		CIFAR-10				NUS-WIDE			
		12 bits	24 bits	32 bits	48 bits	12 bits	24 bits	32 bits	48 bits
Deep methods	Ours	**0.770**	**0.774**	**0.788**	**0.783**	**0.791**	**0.828**	**0.832**	**0.836**
	DTSH	0.710	0.750	0.765	0.774	0.773	0.808	0.812	0.824
	DPSH	0.713	0.727	0.744	0.757	0.752	0.790	0.794	0.812
	DQN	0.554	0.558	0.564	0.580	0.768	0.776	0.783	0.792
	DHN	0.555	0.594	0.603	0.621	0.708	0.735	0.748	0.758
	NINH	0.552	0.566	0.558	0.581	0.674	0.697	0.713	0.715
	CNNH	0.439	0.511	0.509	0.522	0.611	0.618	0.625	0.608

Nondeep methods	FastH	0.305	0.349	0.369	0.384	0.621	0.650	0.665	0.687
	SDH	0.285	0.329	0.341	0.356	0.568	0.600	0.608	0.637
	KSH	0.303	0.337	0.346	0.356	0.556	0.572	0.581	0.588
	LFH	0.176	0.231	0.211	0.253	0.571	0.568	0.568	0.585
	SPLH	0.171	0.173	0.178	0.184	0.568	0.589	0.597	0.601
	ITQ	0.162	0.169	0.172	0.175	0.452	0.468	0.472	0.477
	SH	0.127	0.128	0.126	0.129	0.454	0.406	0.405	0.400

**Table 6:** MAP results for different numbers of bits on the MS-COCO dataset.

Methods	MS-COCO			
	8 bits	16 bits	24 bits	32 bits
Ours	**0.761**	**0.765**	**0.768**	**0.772**
DQN	0.649	0.653	0.666	0.685
DHN	0.607	0.677	0.697	0.701
CNNH	0.505	0.564	0.569	0.574
SDH	0.541	0.555	0.560	0.564
KSH	0.492	0.521	0.533	0.534
ITQ-CCA	0.501	0.566	0.563	0.562

## Data Availability

Data are owned by a third party: the experimental part of this paper uses three widely used public datasets (CIFAR-10, MS-COCO, and NUS-WIDE), which can be publicly accessed. CIFAR-10 can be accessed at http://www.cs.toronto.edu/kriz/cifar.html, MS-COCO at http://mscoco.org, and NUS-WIDE at http://lms.comp.nus.edu.sg/research/NUS-WIDE.htm The implementation code of the algorithm in this paper is written by PyTorch. The code can be obtained from Mingyong Li upon request at limingyong@cqnu.edu.cn.
